# Celebrating the American Society of Plant Biologists centennial anniversary: A compendium of review articles in plant biology

**DOI:** 10.1093/plphys/kiae141

**Published:** 2024-03-28

**Authors:** Nancy A Eckardt, Blake C Meyers, Yunde Zhao

**Affiliations:** The Plant Cell, American Society of Plant Biologists, USA; The Plant Cell, American Society of Plant Biologists, USA; UC Davis Genome Center, University of California Davis, Davis, CA 95616, USA; Plant Physiology, American Society of Plant Biologists, USA; Department of Cell and Developmental Biology, University California San Diego, La Jolla, CA 92093-0116, USA

This year we celebrate 100 years of the American Society of Plant Biologists (ASPB; formerly the American Society of Plant Physiologists). A major motivation for the society was the intent to publish a journal devoted to plant physiology (Hanson 1989), and the first issue of *Plant Physiology* was published in January 1926. *The Plant Cell* began publication in January 1989, to fill a gap in the rapidly progressing areas of modern plant molecular cell and developmental biology (Goldberg et al. 2019). To celebrate the 100-year milestone of ASPB and the society's role in the publication of the highest-quality plant science over the ensuing years, we are pleased to present, in this issue of both journals, a compendium of review articles covering a range of topics in plant biology. The list of topics was divided more or less randomly between the 2 journals, and in each case, a more senior author was invited and asked to consider, where feasible, bringing on board one or more early- or mid-career coauthors.

The May issue of *Plant Physiology* includes 11 articles for this compendium. Two articles delve into aspects of plant metabolism. In *The end game(s) of photosynthetic carbon metabolism*, Sharkey (2024) reviews the last stages of photosynthetic metabolism in leaves that occur after the Calvin–Benson cycle, including major discoveries in sucrose and starch synthesis. Dixon and Dickinson (2024) follow with *A century of studying plant secondary metabolism—From “what?” to “where, how, and why?”* describing the fascinating history of research on small molecules known as plant secondary metabolites and proposing future areas of exploration. Five reviews cover topics in plant development and signaling. Maple et al. (2024) bring us *Flowering time: From physiology, through genetics to mechanism* covering flowering time mutants, natural variation in flowering time, and molecular mechanisms that can inform breeding strategies for new crops and changing environments. In *Light signaling in plants—a selective history*, Huq et al. (2024) review the discovery and characterization of phytochrome and cryptochrome sensory photoreceptors. Shani et al. (2024) cover all things gibberellin: biosynthesis, metabolism, perception, signaling, and transport in *Highlights in gibberellin research: A tale of the dwarf and the slender*. Gasperini and Howe (2024) then guide us through the “blurred distinction between classical phytohormones and other bioactive metabolites” using jasmonate as an example in their review *Phytohormones in a universe of regulatory metabolites: Lessons from jasmonate*. Kim et al. (2024) explore the convergence of signaling pathways in plant responses to dehydration and temperature changes in their review *Regulatory networks in plant responses to drought and cold stress*, with an eye toward engineering climate-resilient crops. Two reviews focus on aspects of plant reproduction. Zhong et al. (2024) review major events in plant reproduction, including gametic differentiation to pollen–pistil interaction, fertilization, and early zygotic events in *From gametes to zygote: Mechanistic advances and emerging possibilities in plant reproduction*. In *Molecular basis and evolutionary drivers of endosperm-based hybridization barriers*, Bente and Köhler (2024) focus on the endosperm, which plays a key role in supporting embryo growth and germination in flowering plants. Rounding out the set in *Plant Physiology* are 2 reviews covering topics in cell biology and synthetic biology. In *A charged existence: A century of transmembrane ion transport in plants*, Blatt (2024) brings us into the field of electrophysiology, focusing on stomatal guard cells, with an engaging review of the history and current state of research on ion transport across plant membranes. Finally, in *A pilot oral history of plant synthetic biology*, Joshi and Hanson (2024) provide a fascinating glimpse into the world of plant synthetic biology, revealed through interviews with eight of the pioneering researchers in the field.

The May issue of *The Plant Cell* includes 17 articles for this compendium. Four articles treat topics in genomics and evolution. Paterson and Queitsch (2024) explore the relationship between angiosperm genome organization and botanical diversity in *Genome organization and botanical diversity*, showing how the indeterminate development of plants may have led to some of the major differences in gene regulation between plants and animals. Mabry et al. (2024) seek to extend the usefulness of model species across larger taxonomic groups in their review *Complementing model species with model clades*, focusing on establishing the Brassicales as a model clade. In *Domestication and the evolution of crops: Variable syndromes, complex genetic architectures, and ecological entanglements*, Alam and Purugganan (2024) review major themes in crop domestication research as key to developing resource-efficient crops and sustainable cultivation practices. In *Why should we study plant sex chromosomes?*, Charlesworth and Harkess (2024) review the evolution of dioecious plant species and features of sex chromosomes such as heteromorphism, recombination suppression, dosage compensation, and the repeated evolution and breakdown of dioecy. Two reviews take us into topics in cell biology. Delmer et al. (2024) review major aspects of the 5 major plant cell wall polymers, recognizing “some of the colorful historical figures who shaped cell wall research over the past 100 years” in *The plant cell wall—dynamic, strong, and adaptable—is a natural shapeshifter*. Zhuang et al. (2024) highlight the important technologies and key scientists who contributed to research on plant organelles and the endomembrane system over the last century in *A century journey of organelles research in the plant endomembrane system*. Three reviews cover fundamental topics in plant development. In *Reflections on the ABC model of flower development*, Bowman and Moyroud (2024) review the colorful history of the ABC model of flower development, highlighting “unsolved riddles still hidden in the floral alphabet.” Sajeev et al. (2024) review the complex and interlinked processes of seed dormancy and germination in *A commitment for life: Decades of unraveling the molecular mechanisms behind seed dormancy and germination*. In their review *Not so hidden anymore: Advances and challenges in understanding root growth under water deficits*, Voothuluru et al. (2024) explore biophysical and metabolic constraints affecting the interactions of root systems with the soil environment and plants’ ability to maintain access to soil water under water deficit conditions. Six more articles explore various topics in phytohormones and signaling. In *An auxin research odyssey: 1989–2023*, Cohen and Strader (2024) review this “master regulator” of plant processes, the role of auxin in spatial and temporal regulation of plant growth and development, and the role *The Plant Cell* has played in publishing seminal work in this area. In *Cytokinin: From autoclaved DNA to two-component signaling*, Argueso and Kieber (2024) review cytokinin function, metabolism, transport, and the nature of cytokinin 2-component signaling pathways. Spoel and Dong (2024) review the major plant defense hormone salicylic acid and its crosstalk with other phytohormones in *Salicylic acid in plant immunity and beyond*. Dodds et al. (2024) review some of the major themes in plant immunity in *Pathogen perception and signaling in plant immunity*, including the gene-for-gene hypothesis, nucleotide-binding/leucine-rich-repeat receptor (NLR) receptors, pattern-triggered and effector-triggered immunity, and efforts in NLR engineering. In *Nitrogen sensing and regulatory networks: It's about time and space*, Shanks et al. (2024) focus on temporal and spatial aspects of nitrogen sensing and signaling and discuss efforts to improve nitrogen-use efficiency in crops. Yang et al. (2024) turn to the essential nutrient phosphorus and review the molecular mechanisms that regulate phosphorous starvation responses and phosphorus transport, sensing, and signaling in *Milestones in understanding transport, sensing, and signaling of the plant nutrient phosphorus*. Finally, 2 reviews focus on aspects of transcription and translation. Marathe et al. (2024) explore the understudied area of extra-nuclear transcription factors that reside in the cytoplasm in an inactive state, entering the nucleus and becoming transcriptionally active only in response to specific signals, such as from biotic and abiotic stresses, in *Should I stay or should I go? Trafficking of plant extra-nuclear transcription factors*. Wu et al. (2024) provide a comprehensive yet concise and engaging review of how the translation of messenger RNA is regulated in plants, providing an excellent summary and useful illustrations of this complex topic in *What, where, and how: Regulation of translation and the translational landscape in plants*.

We acknowledge that this collection is far from a complete representation of the major topics in plant biology. Life often gets in the way of the best intentions, and some invited authors were unable to submit in time to make publication in this issue. Other topics were omitted if recently covered, for example, with extensive reviews in recent or upcoming focus issues in either journal (including e.g. many topics within the areas of cell biology, gene editing, RNA biology, plant immunity, photosynthesis, and proteolysis). We apologize if your favorite topic is not included. We thank Brian Larkins for the initial ideas and discussions that led to this project. We hope our readers find these articles instructive and inspirational as avenues into the next 100 years of plant biology research.
